# Effects of age and sex on vasomotor activity and baroreflex sensitivity during the sleep–wake cycle

**DOI:** 10.1038/s41598-022-26440-3

**Published:** 2022-12-27

**Authors:** Chia-Hsin Yeh, Terry B. J. Kuo, Jia-Yi Li, Kuan-Liang Kuo, Chang-Ming Chern, Cheryl C. H. Yang, Hsin-Yi Huang

**Affiliations:** 1 Institute of Brain Science, National Yang Ming Chiao Tung University, No. 155, Sec. 2, Li-Nong St., Beitou, Taipei, 11221 Taiwan; 2 Sleep Research Center, National Yang Ming Chiao Tung University, Taipei, Taiwan; 3 Center for Mind and Brain Medicine, Tsaotun Psychiatric Center, Ministry of Health and Welfare, Nantou, Taiwan; 4 Brain Research Center, National Yang Ming Chiao Tung University, Taipei, Taiwan; 5 Department of Health and Leisure Management, Yuanpei University of Medical Technology, Hsinchu, Taiwan; 6 Department of Family Medicine, Taipei City Hospital Renai Branch, Taipei, Taiwan; 7 Institute of Biomedical Informatics, National Yang Ming Chiao Tung University, Taipei, Taiwan; 8 Division of Neurology, Taipei Veterans General Hospital, Taipei, Taiwan; 9 Department of Neurology, En Chu Kong (ECK) Hospital, 399 Fu-Xing Road, Sanxia District, New Taipei City, Taiwan 23702; 10 Department of Education and Research, Taipei City Hospital, Taipei, Taiwan; 11 Information Management Office, Taipei Veterans General Hospital, Taipei, 112 Taiwan; 12 Institute of Clinical Medicine, National Yang Ming Chiao Tung University, Taipei, 112 Taiwan

**Keywords:** Cardiology, Neurology

## Abstract

Cardiovascular function is related to age, sex, and state of consciousness. We hypothesized that cardiovagal baroreflex sensitivity (BRS) demonstrates different patterns in both sexes before and after 50 years of age and that these patterns are associated with patterned changes during the sleep–wake cycle. We recruited 67 healthy participants (aged 20–79 years; 41 women) and divided them into four age groups: 20–29, 30–49, 50–69, and 70–79 years. All the participants underwent polysomnography and blood pressure measurements. For each participant, we used the average of the arterial pressure variability, heart rate variability (HRV), and BRS parameters during the sleep–wake stages. BRS and HRV parameters were significantly negatively correlated with age. The BRS indexes were significantly lower in the participants aged ≥ 50 years than in those aged < 50 years, and these age-related declines were more apparent during non-rapid eye movement sleep than during wakefulness. Only BRS demonstrated a significantly negative correlation with age in participants ≥ 50 years old. Women exhibited a stronger association than men between BRS and age and an earlier decline in BRS. Changes in BRS varied with age, sex, and consciousness state, each demonstrating a specific pattern. The age of 50 years appeared to be a crucial turning point for sexual dimorphism in BRS. Baroreflex modulation of the cardiovascular system during sleep sensitively delineated the age- and sex-dependent BRS patterns, highlighting the clinical importance of our results. Our findings may aid in screening for neurocardiac abnormalities in apparently healthy individuals.

## Introduction

The cardiovascular system is controlled by the baroreflex and central autonomic brain regions^1^. Baroreflex sensitivity (BRS) is considered an index of cardiac autonomic regulation in humans; it comprises cardiovagal and sympathetic components, which are responsible for both short- and long-term blood pressure regulation^2^. In general, BRS and arterial pressure variability (APV) are crucial mechanisms underlying the neural regulation of the cardiovascular system^3^. Typically, daily activities are related to the state dependency of the patterned responses of cardiovascular function. For instance, the sleep–wake cycle is related to stable arterial pressure maintenance and pattern differences in the cardiovascular system^4^. We previously reported that differences in cardiovascular variabilities between Wistar–Kyoto rats (WKYs) and spontaneously hypertensive rats (SHRs) were more evident during sleep; BRS tended to be significantly higher in the WKYs during sleep but was nonsignificant in the SHRs^5,6^. These results are similar to those of a human study indicating that BRS is higher during non-rapid eye movement sleep (nREM) than during wakefulness^7^. However, due to the lack of relevant comprehensive studies, the effects of basic physiological factors on BRS remain unclear.

Cardiovascular events may be related to both circadian variations and the sleep–wake cycle^8^. The sympathetic BRS acts as a buffer that dampens surges in sympathetic activation by rapidly changing cardiac vagal circuits throughout the overnight sleep period^9^. Therefore, BRS is an essential factor in humans during sleep. Recently, a population-based study confirmed that age and sex were important factors associated with BRS^10^; however, research on BRS in both the sexes of different ages during different consciousness states remains scant. Nevertheless, the CNS-ANS coupling is different among the conscious states^11^. Only analyzing the awake state cannot fully reflect the impact of biological variables on BRS. In our previous animal study, SHRs demonstrated increased sympathetic modulation during sleep; however, this sympathetic modulation was less evident in SHRs who were awake^12^. Measuring HRV and BRS indexes during sleep can have considerable implications in research on neurocardiac events. However, few studies have investigated the effects of physiological factors on neural cardiovascular regulation during sleep, particularly using spontaneous BRS methods.

In our previous studies, we observed age-dependent differences in HRV; for instance, postmenopausal women had lower high-frequency of HRV (HF) but higher low-frequency of HRV (LF) percentage and LF/HF ratio than did young premenopausal women^13^. Moreover, normal aging led to declines in both cardiac parasympathetic nerve activity^14^ and sympathetic BRS^15^. Autonomic activity prominently declined before the age of 50 years^16^. However, the effects of age and sex on low-frequency power of APV (BLF) and BRS have not been reported thus far. In particular, age-related changes in BRS remain unclear, possibly because studies conducted thus far have included ethnically different populations, a small sample size, or only a few age groups.

We previously investigated the effects of sex on the sympathetic and parasympathetic control of HR. The results revealed that sex-related differences in parasympathetic regulation were not observed after the age of 50 years, whereas the dominance of sympathetic regulation diminished later in men than in women^17^. Notably, estrogen is a major factor controlling sex-related autonomic differences^13^. The rate of the BRS decline throughout the entire lifespan was similar for both sexes^18^. In addition, menopause was noted to be a crucial time point, where BRS impairment is observed^19^. Moreover, the age of 50 years appeared to be a crucial turning point for physiological and psychological functioning in both sexes^16,17^. However, few studies have investigated sex-related differences in BRS among all age groups under different consciousness states.

On the basis of the aforementioned findings, we hypothesized that in both sexes, cardiovagal BRS demonstrates different patterns before and after the age of 50 years. Moreover, the different patterns are associated with patterned changes during the sleep–wake cycle. Therefore, the current study investigated the effects of age and sex on blood pressure (BP), APV, and BRS during the states of wakefulness and sleep and compared age and sex-related pattern differences in BP, APV and BRS between wakefulness and sleep.

## Methods

### Participants

In total, 67 healthy volunteers aged 20 to 79 years (41 women) were recruited through an online advertisement. We believe that special consideration should be given to the menopausal and andropausal interval in the age variable in order to more accurately detect the impact of sex on BRS, so we use 50 years as the cutpoint for subject grouping^20–22^. All of them had a regular sleep–wake pattern (i.e., night sleep) and did not consume any medication, alcohol, caffeine, or nicotine. Body mass index (BMI) of all participants were in the normal range of 18.5–25 kg/m^2^. None of the participants had a reported medical history of psychiatric, neurological, and cardiovascular illnesses. Moreover, they did not demonstrate any signs of substance abuse or sedative or hypnotic drug use.

### Experimental procedures and data recording

Initially, all the participants were divided into 4 age groups: 20–29, 30–49, 50–69, and 70–79 years. All the participants underwent laboratory polysomnography (PSG) at noon and BP recording for 1 h. Here, we used standard PSG measurements (Embla SX, Natus Medical Incorporated, USA) to assess sleep cycle. Brain waves were recorded at four reference points (C3-A2, C4-A1, O1-A2, and O2-A1) by electroencephalogram (EEG) while eye movements were recorded through electrooculography. Chin muscle tone was measured through electromyography (chin-EMG), and cardiac activity was recorded by electrocardiogram (ECG) at the V5 site on the chest. Continuous BP measurements were obtained using Finometer PRO (Finapres Medical Systems, Amsterdam, Netherlands). For finger blood calibration, a 2-min baseline recording was performed using an upper arm cuff before continuous recording was implemented during sleep. Real-time BP signals were synchronized using the PSG software program Somnologica (Embla, Inc., Denver, CO).

Sleep stages, namely wakefulness, rapid eye movement sleep (REM), and nREM, were scored in accordance with standard criteria described elsewhere^16,23^.

### Signal processing and data analysis

Power spectral analysis was used for the HRV frequency domain measurement. The stationary R-R interval (RR) signal was curtailed into successive 64-s (4096 point) time segments (i.e., windows or epochs) with 50% overlap for analysis. Analytical procedures used here for HRV analysis have been detailed elsewhere^12,24,25^. Through Fourier transformation, the spectral information of HRV was classified into total power (TP), HF (0.15–0.4 Hz), LF (0.04–0.15 Hz), LF/HF ratio, and normalized LF (LF%) of the RR spectrogram were enumerated for each time segment. RR and TP are related to both sympathetic and parasympathetic nervous systems^17^. HF can reflect vagal modulation, and LF% and LF/HF are considered to be sympathetic modulation markers or represent the sympathovagal balance^26^.

For sleep stage analysis, we performed computerized sleep analysis in line with the criteria defined by Rechtschaffen and Kales and the American Academy of Sleep Medicine^16,27^. All the obtained results were verified by a qualified sleep technician. All PSG data were manually scored in 30-s epochs for each consciousness state including wakefulness and nREM.

We designed a special program in the Pascal language (Borland Pascal 7.0, Borland, USA) for analyzing our EEG and ECG data; ECG signals were preprocessed in accordance with recommended procedures^28^ as indicated in our previous studies^17,29^. In brief, both ventricular premature complexes and artifacts were identified and the QRS complex was acquired using a computer algorithm. For the continuity in the time domain, resampling and linear interpolation were applied at a rate of 64 Hz to RR. Moreover, the sampling rate was adjusted to 64 Hz for all EEG signals. Power spectral analysis was performed to measure EEG, APV, and HRV amplitudes. EEG, arterial pressure, and RR signals were truncated into successive 64-s (4096 points) time segments (windows or epochs) with 50% overlap. In addition, the Hamming window was applied^24^. Subsequently, the power density of the spectral components was estimated on the basis of fast Fourier transformation, and attenuation originating from sampling was corrected; the Hamming window was also implemented here^17^.

For APV and HRV analysis, we used a previously reported methodology^5,12,17,25,26^. In brief, the mean AP (MAP) and mean RR were estimated from the digitized AP and ECG signals, respectively. In addition, resampling and interpolation were performed to provide continuity in the time domain. Fast Fourier transformation and the Hamming window were used for these sequences^24^. Either HF (0.15–0.4 Hz) or LF% (0.04–0.15 Hz) was quantified from the RR spectrogram. BLF (0.04–0.15 Hz), a marker of sympathetic vasomotor control, and the high-frequency power of APV (BHF) was quantified from the AP spectrogram from each time segment^25,26^.

Spontaneous BRS was derived from the AP–RR transfer function and AP–RR linear regression^25,26^. In brief, the transfer magnitude at HF (BrrHF) and LF (BrrLF) ranges were estimated using transfer function analysis. For linear regression analysis, the ascending and descending slopes of AP and RR pairs were defined as BrrA and BrrD, respectively. Finally, APV, HRV, and BRS during the sleep–wake state were averaged for each participant.

### Statistical analysis

Continuous data (HF, LF%, LF/HF, BP, BLF, BHF, BrrLF, BrrHF, BrrA, and BrrD) are presented as medians (min–max), whereas categorical data (sex and age groups) are presented as numbers (percentages).

We used the Kruskal–Wallis test followed by the Mann–Whitney U test with Bonferroni adjustment to determine between-group differences. The relationship between parameters was analyzed using Spearman correlation analysis. We next performed linear regression analysis to confirm the association among BP, APV, BRS, and age. All statistical analyses were performed using SPSS (version 17; SPSS, Chicago, IL, USA). In general, *p* < 0.05 was assumed to indicate statistical significance. However, in post hoc analysis, *p* < 0.008 was assumed to indicate significance.

### Ethical approval

All the participants provided written informed consent for participation after the experimental procedures had been described to them. The procedures used in this study were approved by the Institutional Review Board of Taipei Veterans General Hospital (approval number: 1000057). All research was performed in accordance with relevant guidelines.

Our research is classified in observational studies which don't test potential treatments; instead we observe participants and track health outcomes over time. We followed the ICMJE (INTERNATIONAL COMMITTEE of MEDICAL JOURNAL EDITORS) definition of clinical trial: Purely observational studies (those in which the assignment of the medical intervention is not at the discretion of the investigator) will not require registration.

## Results

### Basic characteristics and HRV and BRS indexes among different age group

Table 1 summarizes the demographic characteristics of the included 67 patients. No significant differences in BMI, systolic BP, and diastolic BP were noted among the different age groups (Table 1).

**Table 1:** Demographic Characteristics of the Different Age Groups.

Demographics (yrs)	20–29	30–49	50–69	70–79	*p*
Number	n = 19	n = 23	n = 18	n = 7	0.558
Sex (male/female)	(9/10)	(8/15)	(5/13)	(3/4)	
BMI (kg/m^2^)	21.17 ± 2.34	23.09 ± 3.21	22.11 ± 2.41	23.12 ± 4.03	0.267
SBP (mmHg)	112.32 ± 10.21	115.05 ± 12.65	118.50 ± 10.74	125.57 ± 15.55	0.169
DBP (mmHg)	69.68 ± 6.33	77.00 ± 9.05	76.07 ± 10.62	79.86 ± 10.76	0.064

Mean ± SD.

BMI, body mass index; SBP, systolic blood pressure; DBP, diastolic blood pressure.

Chi-square test and Kruskal–Wallis test were used.

The analysis of HRV indexes among the different age groups (Table S1) revealed the correlation of autonomic function with age and consciousness states; this finding is consistent with that of our previous study^16^. In general, BRS indexes were more strongly and inversely correlated with age compared with HRV indexes (Table S2).

### BP/APV profiles and correlation coefficients between BP/APV and age in all participants under different consciousness states

**Figure 1:**
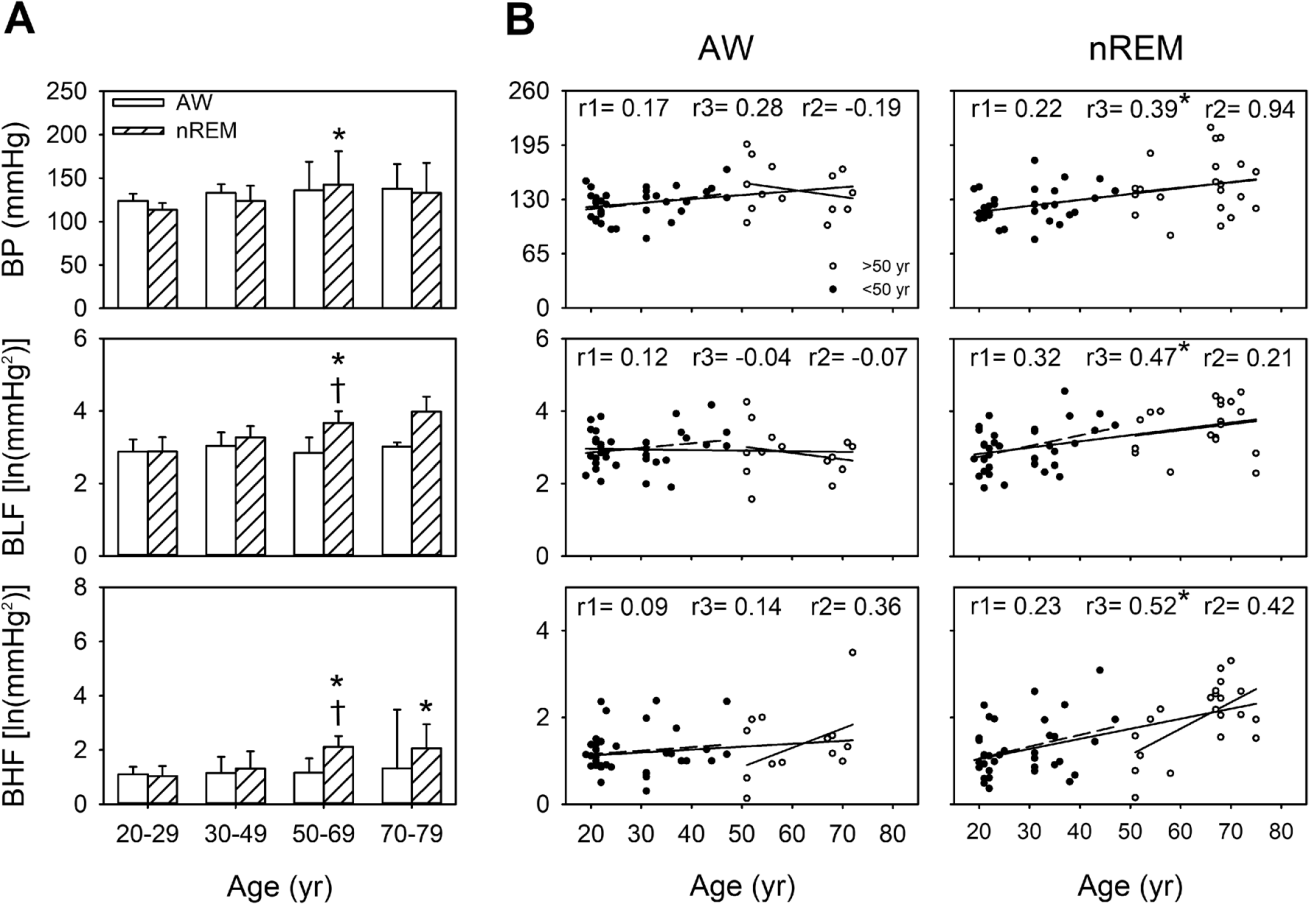
Blood pressure (BP)/arterial pressure variability (APV) parameters during wakefulness (AW) and non-rapid eye movement sleep (nREM) in the different age groups, and the comparisons of BP/APV between the AW and nREM for the different age groups in all participants (**A**). *p < 0.05 vs. 20–29. †*p* < 0.05 vs. the awake stage. The values are presented as means ± SEMs. Two-dimensional scattergrams for the BP/APV–age relationship during AW and nREM in all participants (**B**). *p < 0.05. r1: correlation coefficient in the 20–49-year age group. r2: correlation coefficient of 50–79-year age group. r3: correlation coefficient of 20–79-year age group. Abbreviations: ln, natural logarithm; BLF, LF of arterial pressure variability.

**Figure 2:**
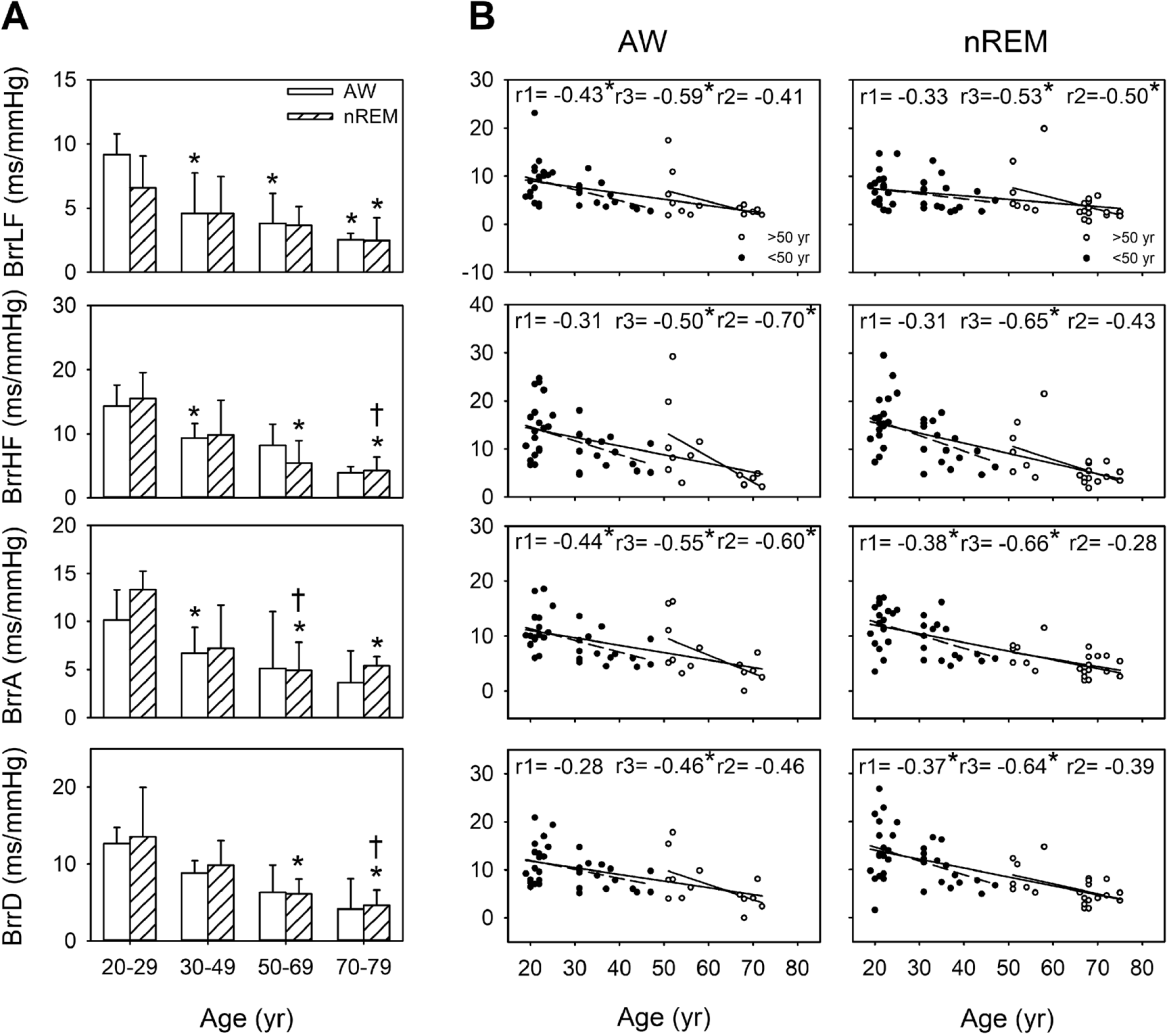
Baroreflex sensitivity (BRS) parameters during wakefulness (AW) and non-rapid eye movement sleep (nREM) in the different age groups in all participants and their comparison for different consciousness states (**A**). *p < 0.05 vs. 20–29-year age group. †*p* < 0.05 vs. 30–49-year age group. The values are presented as means ± SEMs. Two-dimensional scattergrams for the BRS–age relationship during AW and nREM in all participants (**B**). *p < 0.05. r1: correlation coefficient in the 20–49-year age group. r2: correlation coefficient of 50–79-year age group. r3: correlation coefficient of 20–79-year age group. Abbreviations: BrrHF/BrrLF, magnitude of the MAP–R-R interval transfer function; BrrA/BrrD, slopes of MAP–R-R interval linear regression.

During wakefulness, BP and BHF tended to increase with age. During nREM, compared with the individuals aged 20–29 years, BP, BLF, and BHF were significantly elevated in the individuals aged 50–69 years (*p* = 0.004, 0.001, and 0.006, respectively), whereas only BHF was significantly elevated in the individuals aged 70–79 years (*p* = 0.004). In all the participants in this study (aged 20–79 years), the results of simple linear regression indicated that BP was positively correlated with age in both the AW and nREM stages. However, this correlation was stronger during nREM. In contrast to BP, BLF and BHF significantly differed among different consciousness states in the individuals aged 50–69 years. Similarly, age demonstrated a moderately positive correlation with BLF (r = 0.47, *p* < 0.05) and BHF (r = 0.52, *p* < 0.05) during nREM but not during wakefulness. Notably, the correlation between BP and age was stronger during nREM (r = 0.39, *p* < 0.05). Furthermore, changes in sympathetic activity with normal aging were noted in the individuals aged 50–69 years only during nREM. We noted that BLF, BHF, and HF were moderately correlated with age (Fig. 2; Table S2); specifically, the BLF–age and BHF–age correlation was positive, but the HF–age correlation was negative (Fig. 2; Table S2). These findings indicated the importance of sympathetic activity and BP during sleep (Fig. 1).

### BRS profiles and correlation coefficients between BRS and age in all participants under different consciousness states

Before the age of 50 years, BRS was higher during nREM than during wakefulness. During wakefulness, significantly lower BRS indexes (i.e., BrrLF, BrrHF, and BrrA) were observed in the individuals aged 30–49 years (*p* = 0.006, 0.007, and 0.002, respectively) compared with the individuals aged 20–29 years. Moreover, compared with the individuals aged 20–29 years, those aged 50–69 and 70–79 years demonstrated significantly lower BrrLF (*p* = 0.008 and 0.006, respectively). Among sleep-related BRS measurements, BrrHF, BrrA, and BrrD were significantly lower in the individuals aged 50–69 years (all *p* < 0.001) and 70–79 years (*p* = 0.001, 0.003, and 0.003, respectively) than in those aged 20–29 years. Moreover, BrrHF and BrrD were significantly lower in the individuals aged 70–79 years (*p* = 0.006 and 0.008, respectively) than in those aged 30–49 years. BrrA was significantly lower in the individuals aged 50–69 and 70–79 years than those aged 20–29 years (*p* = 0.001 and 0.003, respectively), and it was significantly lower in the those aged 50–69 years than in those aged 30–49 years (*p* = 0.007); moreover, BrrLF was significantly lower in the individuals aged 70–79 years than in those aged 20–29 years (*p* = 0.005). Compared with BLF (r = 0.47, *p* < 0.001), the BRS parameters (i.e., BrrLF, BrrHF, BrrA, and BrrD) demonstrated a negative correlation with age (r =  − 0.53, *p* < 0.001; r =  − 0.65, *p* < 0.001; r =  − 0.66, *p* < 0.001; r =  − 0.64, *p* < 0.001) during nREM. Significant negative correlations were observed between BrrLF and age among all the age groups (r =  − 0.53, *p* < 0.001) and in the 50–79-year age group (r =  − 0.50, *p* = 0.020); however, this correlation was not observed in the 20–49-year age group. Moreover, BrrA (r =  − 0.38, *p* = 0.023) and BrrD (r =  − 0.37, *p* = 0.028) demonstrated a more considerable decline in the earlier life stages than did BrrLF. Moreover, the age-related decline in BRS (i.e., BrrLF, BrrHF, BrrA, and BrrD) was more apparent during nREM (r =  − 0.53, − 0.65, − 0.66, and r =  − 0.64, respectively; all *p* < 0.001) than during AW (r =  − 0.59, *p* < 0.001; r =  − 0.50, *p* < 0.001; r =  − 0.55, *p* < 0.001; and r =  − 0.46, *p* = 0.001, respectively). Therefore, BRS indexes during sleep are a vital indicator of the effect of age on BRS (Fig. 2).

### Comparison of BP/APV between sexes during different consciousness states across different age groups

**Figure 3:**
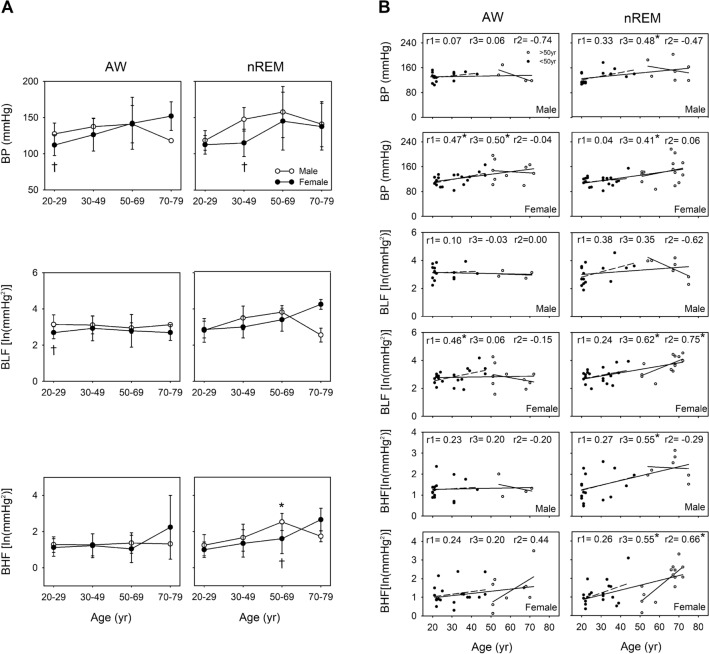
Comparison of blood pressure (BP)/arterial pressure variability (APV) between wakefulness (AW) and non-rapid eye movement sleep (nREM) for the different age groups in men and women (**A**). * *p* < 0.05 vs. 20–29-year age group. †*p* < 0.05 vs. men. The values are presented as means ± SEMs. Two-dimensional scattergrams for the BP/APV–age relationship during AW and nREM in men and women (**B**). *p < 0.05. r1: correlation coefficient in the 20–49-year age group. r2: correlation coefficient of 50–79-year age group. r3: correlation coefficient of 20–79-year age group. Abbreviations: ln, natural logarithm; BLF, LF of arterial pressure variability.

Changes in BP with aging were more notable during nREM than during wakefulness. A significant correlation was noted between BP and age both during wakefulness and nREM in women (r = 0.50, *p* = 0.006 and r = 0.41, *p* = 0.018, respectively); however, this correlation was noted only during nREM in men (r = 0.48, *p* = 0.020). If we examine all the participants aged 20–79 years, BP during nREM was observed to have a moderate to strong relationship with age both in the men and women (r = 0.48, *p* = 0.020 and r = 0.41, *p* = 0.018, respectively). In addition, the men aged 20–49 years were found to have significantly higher BP than did the women during wakefulness (*p* = 0.041) and nREM (*p* = 0.005). During nREM, BLF was significantly and positively correlated with age in the women aged 20–79 years and those aged 50–79 years (r = 0.62, *p* < 0.001 and r = 0.75, *p* = 0.002, respectively); however, this result was not observed in the men. During wakefulness, significant sex differences were noted. Among the individuals aged 20–29 years, BLF was higher in the men than in the women (*p* = 0.037). During nREM, BHF was positively correlated with age among the women (r = 0.55, *p* = 0.001) and men (r = 0.55, *p* = 0.007) of all ages and in the women aged 50–79 years (r = 0.66, *p* = 0.011). Finally, significant differences were observed in BHF between the men and women in the 50–69-year group (*p* = 0.036) only during nREM (Fig. 3).

### Comparison of BRS and age between sex under different consciousness states across different age groups

**Figure 4:**
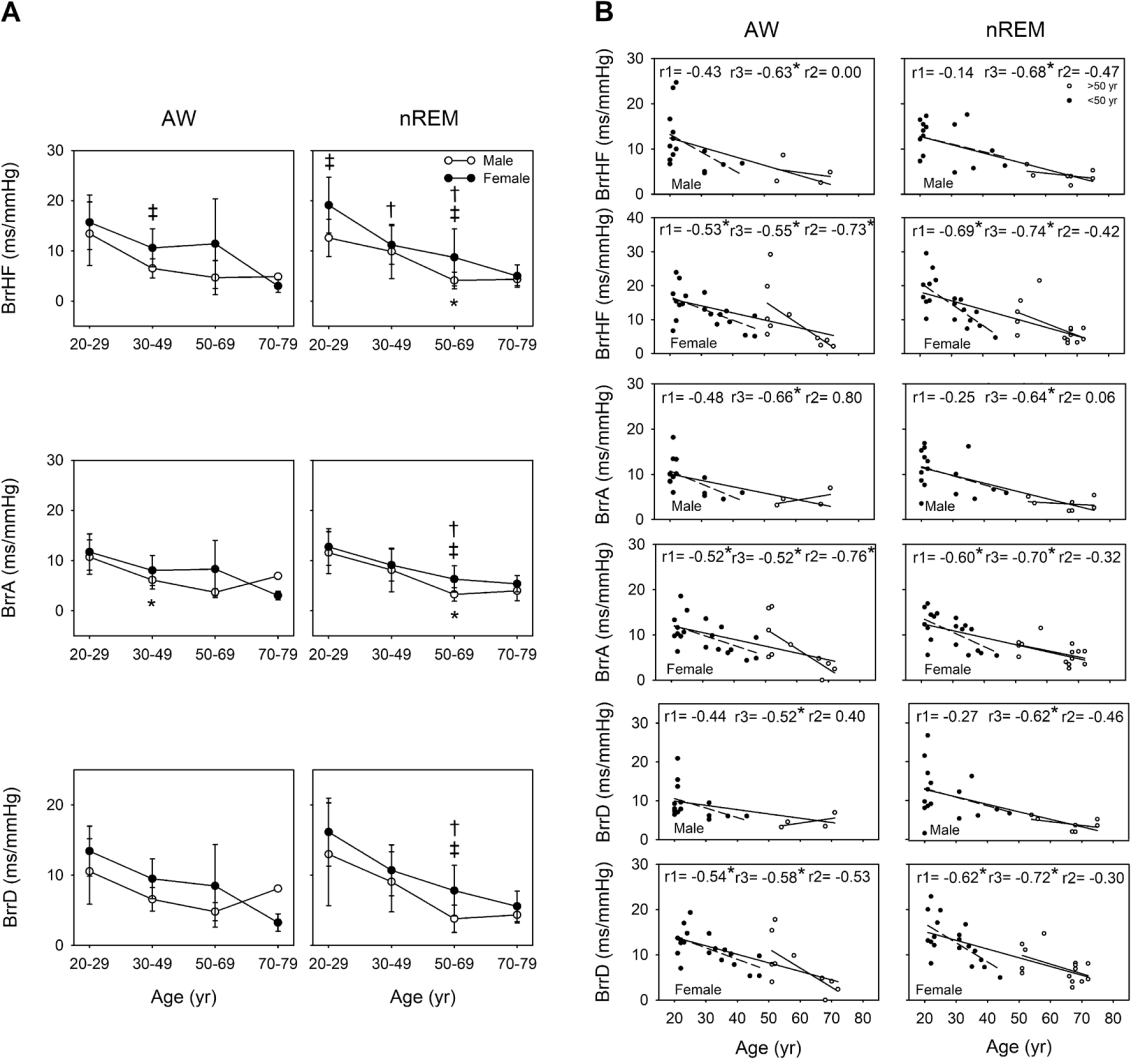
Comparison of baroreflex sensitivity (BRS) between wakefulness (AW) and non-rapid eye movement sleep (nREM) for the different age groups in men and women (**A**). *p < 0.05 vs. 20–29-year-old men. †*p* < 0.05 vs. 20–29-year-old women. ‡ *p* < 0.05 vs. men. The values are presented as means ± SEMs. Two-dimensional scattergrams for the BRS–age relationship during AW and nREM in men and women (**B**). *p < 0.05. r1: correlation coefficient in the 20–49-year age group. r2: correlation coefficient of 50–79-year age group. r3: correlation coefficient of 20–79-year age group. Abbreviations: ln, natural logarithm; BrrHF, magnitude of MAP–R-R interval transfer function; BrrA/BrrD, slope of MAP–R-R interval linear regressions.

The BRS pattern along with aging was similar during wakefulness to that during nREM. Our simple linear regression analysis results indicated that aging was associated with a decline in BRS in both the men and women. Moreover, a relatively significant decline was noted in BrrA and BrrD among the 20–49-year-old women during wakefulness (r =  − 0.52, *p* = 0.023 and r =  − 0.54, *p* = 0.017, respectively) and nREM (r =  − 0.60, *p* = 0.007 and r =  − 0.62, *p* = 0.005, respectively). During nREM, the tendency for the attenuation of BrrHF, BrrA, and BrrD demonstrated a highly negative correlation in both the men (r =  − 0.68, *p* < 0.001; r =  − 0.64, *p* = 0.001; and r =  − 0.62, *p* = 0.002, respectively) and women (r =  − 0.74, *p* < 0.001; r =  − 0.70, *p* < 0.001; r =  − 0.72, *p* < 0.001, respectively). The correlation coefficient was higher in the women than in the men. Moreover, during nREM, compared with the men, the women demonstrated a significantly higher BrrHF in the 20–29-year age group (*p* = 0.007) and significantly higher BrrHF, BrrA, and BrrD in the 50–69-year age group (*p* = 0.041, 0.027, and 0.020, respectively; Fig. 4).

## Discussion

In this study, we determined various HRV and APV parameters during different consciousness states in the 20–79-year-old individuals to evaluate the spontaneous effects of sex, age, and consciousness states on cardiac sympathetic and parasympathetic activity and BRS. By performing AP–RR transfer function and AP-RR linear regression analyses, we quantified changes in spontaneous BRS to investigate relationships between the activities of autonomic nervous system (ANS) and central nervous system (CNS), which control the heart and blood vessels. The major finding of this study is that the central effect on BRS is different before and after the age of 50 years in both sexes; here, the age of 50 years appeared to be the cutoff point. Our findings indicated a stronger correlation between circulation system function and aging in the women than in the men. In addition, degeneration in BRS with age was observed more clearly in the nREM stage than during wakefulness.

Each consciousness state demonstrates unique behavioral and physical patterns owing to specific underlying neurophysiologic mechanisms^30^. Of these, sleep exerts a larger effect on physiological regulation; specifically, physiological changes related to motor control, BP, HR, and respiratory activity occur during sleep, through an antagonistic effect between sympathetic and parasympathetic cardiovascular regulation^31^. The central neural pathways related to autonomic commands underlie the features of BRS during nREM; in other words, the baroreflex resetting toward the lower values of heart rate (HR), BP, and sympathetic nerve activity leads to higher BRS during nREM than during wakefulness. These phenomena possibly illustrate the mechanism underlying CNS–ANS coupling during sleep^11^. In a previous study, WKYs demonstrated significant BRS enhancement during sleep, whereas SHRs exhibited enhanced sympathetic vasomotor activity but attenuated BRS; however, these phenomena were not observed under wakefulness^5,6^. An age-related decline in autonomic functioning was observed in individuals aged > 29 years^16^.

The circadian rhythmicity of the functional regulation of the cardiovascular system is unequivocal^32^. Nocturnal cardiovascular events tend to have a bimodal distribution, leading to their more frequent occurrence at the beginning and end of the night^33,34^. In susceptible patients, the risk of numerous adverse cardiac events, such as arrhythmia, acute myocardial infarction, and sudden cardiac death, are higher during REM, whereas that of ischemic events is higher during nREM^32,35^. We previously observed that changes in sympathetic vasomotor activity and BRS were associated with alterations in the sleep–wake cycle^5^. These findings are corroborated by the current findings regarding sleep-related changes in BP, BLF, BHF and BRS (Figs. 1 and 2). Notably, recordings obtained during nREM highlighted the patterns of BP, APV and BRS, all of which were affected by aging. These changes before and after the age of 50 years were found to be different. As such, cardiovascular vulnerability during sleep is a major factor in susceptible patients.

Similar to the different degeneration pattern of autonomic functioning, which declined rapidly before the age of 50 years and relatively slow after it^16^, our current findings revealed the turning point of BLF and BHF to be approximately the age of 50 years; BLF and BHF demonstrated a considerable increase after 50 years of age (Fig. 1). This increase in sympathetic activity may be related to cardiovascular problems during middle age—the age range in which the apparent attenuation of BRS occurs^36^.

Previous research pointed out normotensive premenopausal women show a vagal predominance of cardiac autonomic modulation, whereas age-matched men show a predominance of sympathetic modulation^37^. Another related research divided the subjects into three age groups (< 30 yrs, 30–60 yrs, and > 60 yrs), and found that there were significant statistical differences in BRS among the three groups^38^. The average age of menopause is around 50 in the Asian and the United States^20,21^. Besides, andropause should also be taken into consideration^22^. Thus we believe that special consideration should be given to the menopausal interval in the age variable in order to more accurately detect the impact of sex on BRS, so we use 50 years as the cutpoint.

Our findings regarding the age-dependent changes were more obvious in BRS than in HRV and APV in the 20–49-year age group, particularly during sleep (Table S2). These findings indicated that the significant degeneration in the ANS may account for the apparent decline in BRS before the age of 50 years, whereas the increase in cardiac sympathetic modulation and sympathetic-driven peripheral vasomotor responses occurred only after the age of 50 years.

Before the age of 50 years, BP and BLF were significantly lower in the women than in the men (Fig. 3), possibly because of the strengthening of β-adrenergic receptor–mediated dilatation and circulating female sex hormones in younger women^39^. Our results are consistent with those reported by Matsukawa et al.: at the age of < 50 years, muscle sympathetic nerve activity was lower in women than in men, but these levels became similar between the sexes after the age of 50 years^40^. In this study, although BP was higher in the men than in the women before the age of 50 years, APV increased in women after the age of 50 years. According to our previous findings, cardiac sympathetic regulation could be evaluated based on respiratory-related APV^41,42^. Moreover, in anaesthetized and positive pressure–ventilated rats, graded hemorrhage was noted to accompany an increase in respiratory-related APV in correlation with autonomic function, particularly β-adrenoceptors^43^. Across all age groups (20–79 years), we found that BLF was positively correlated with age only in the women. Moreover, BLF and BHF were positively correlated with age only in the women, with a sharp increase in their values after the age of 50 years. This observation was noted only during sleep (Fig. 3). The current findings are in line with the observation that the incidence of hypertension is considerably higher around the age of menopause in women and that these this incidence may be identical to or even higher than that in men of the same age group^44^.

Recently, a population-based study confirmed that age and sex were important factors associated with BRS^10^. However, no studies have been found to discuss the impact of biological variables on BRS for sleep–wake cycle. Nevertheless, the CNS-ANS coupling is different in the awake and sleep stages^11^, only the single awake state cannot fully reflect the impact of biological variables on BRS.

Sexual dimorphism has been noted in BRS function. Considerable variabilities, particularly those related to sex differences, lead to beat-to-beat vascular transduction^45^. Studies have reported inconsistent results related to sex differences in BRS among young individuals^46–48^. Nonetheless, around middle age, women have been noted to have higher arterial stiffness than men do^18^. Animal studies have confirmed the negative consequences of the oscillation and deprivation of ovarian hormones on BRS, which is involved in cardiovascular autonomic control, as indicated by changes in arterial pressure^49^. In addition, estrogen treatment significantly increased BRS in ovariectomized rats; however, the β-adrenergic receptor was found to not be involved in this effect^50^. A prospective clinical study reported that a reduction in estradiol levels leads to decreased carotid-vasomotor BRS^51^. In an animal study examining whether testosterone can facilitate baroreflex responsiveness, baroreflex was measured in sham-operated rats and castrated rats treated with phenylephrine and sodium nitroprusside, respectively. The results indicated that decreased plasma testosterone levels attenuated reflex bradycardia in the castrated rates compared with the sham-operated rats^52^. Another study demonstrated obvious cardiovascular changes following the administration of testosterone alone in adolescent rats; these changes included increased arterial pressure, decreased HR, and exacerbated tachycardiac baroreflex response^53^. In the current study, the men demonstrated significantly lower BRS at the age of 50–69 years than did the women during sleep (Fig. 4); this result is in contrast to that of a previous study, which reported that BRS was attenuated in middle-aged women but not in middle-aged men^54^. The possible reason for this inconsistency is as follows: the previous study used the Valsalva method to detect BRS, whereas we measured BRS spontaneously. Moreover, we divided our patients into different age groups and detected their parameters at different consciousness states. Notably, our findings revealed that the women exhibited attenuation in BRS before the age of 50 years; however, the women aged 20–69 years had higher BRS than did the men aged 20–69 years (Fig. 4). This phenomenon was associated with parasympathetic reduction in cardiac parasympathetic activity; however, BRS weakened with age at a relatively slow rate in the men. In addition, all the men demonstrated a significant negative correlation between age and BRS during both nREM and wakefulness, but they did not demonstrate significant differences before and after the age of 50 years (Fig. 4). Autonomic status, BRS, and hormone status have been demonstrated to be correlated, indicating that the lower levels of circulating androgens and estrogens accompany lower HRV and decreased BRS^55^. Thus, both the men and women demonstrate a physiological turning point around middle age; however, this reduction in hormone production appears to be slower in men than in women. The above results are consistent with our hypothesis that the trend of BRS changes with age for men and women before and after the age of 50 is different.

### Perspective and significance

In summary, the aforementioned results supported the hypothesis that when considering sex differences, the patterns of APV and BRS are different across an individual’s lifespan. Around middle age, individual differences in APV and BRS demonstrated considerable variations among the women (Figs. 3 and 4). These results may be related to menopause: sympathetic activity was determined to be significantly higher in perimenopausal women^56^. Moreover, estrogen may play a crucial role in sex-related autonomic differences^13^. Similarly, in the men, andropause was a likely reason for the marked decrease in BRS in the 50–69-year age group than in the 20–29-year age group (Fig. 4). Evidence suggests a positive interaction between testosterone and cardiomotor vagal activity^52^. Testosterone may be involved, at least partly, in the augmentation of cardiac vagal efferent activity, which may have contributed to the results in our study. The aforementioned results supported the hypothesis that the 50 years of age is a critical turning point in human life. Moreover, ANS activity varies with age, with a turning point around the age of 50 years^16^. Therefore, not only neural and hemodynamic factors but also hormones possibly contribute to age- and sex-related differences in BRS.

### Limitations

This study has few limitations. First, although we excluded individuals diagnosed as having hypertension, sporadic BP fluctuations were still noted in our data. In addition, because we did not perform 24-h BP monitoring in our participants, we could not exclude individuals with altered circadian BP rhythms, such as nondipping and inverted dipping. Second, although we controlled for BMI in each age group, the sex ratio in each group was less controlled for and the number of participants in each subgroup was unequal. Finally, because we did not perform any invasive vascular and hormone measurements, the collected indirect data may have had some deviation from the actual data.

## Conclusions

In the present study, changes in BRS were found to vary with age, sex, and consciousness state, with each exhibiting a specific pattern. The age of 50 years seems to be a crucial turning point for sexual dimorphism in BRS. Baroreflex modulation of the cardiovascular system during sleep can delineate the age and sex–dependent BRS pattern more sensitively, indicating the clinical importance of this result. Age-related cardiovascular function impairment demonstrated sex differences. The measurements used in this study may aid in screening for neurocardiac abnormalities in apparently healthy individuals. Additional studies focused on generating a normative database of a healthy population according to age, sex, and consciousness state are warranted.

## Supplementary Information

Supplementary Information.

## Data Availability

All data generated or analysed during this study are included in this published article [and its supplementary information files].
