# Supplementary material for: Effects of age and sex on vasomotor activity and baroreflex sensitivity during the sleep–wake cycle
Source: Sci Rep. 2022 Dec 27;12:22424. doi: 10.1038/s41598-022-26440-3 (PMC9794808; doi:10.1038/s41598-022-26440-3)
Supplement: Supplementary file 1 — Supplementary Information. [file 41598_2022_26440_MOESM1_ESM.docx]

**Comparison of HRV Among Different Age Groups**

HRV indexes (i.e., RR, HF, LF, LF/HF, and LF%) in different consciousness states stratified by the age group were examined using the Wilcoxon signed-rank test. LF was significantly lower during nREM than during AW in the 20–29-year age group; this trend was also noted for LF/HF and LF% in the 20–29- and 70–79-year age groups. By contrast, in the 30–49-year age group, HF was significantly higher during nREM than during wakefulness (Table S1).

**Correlation of Age With HRV and BRS during Wakefulness and nREM in Different Age Groups**

The effects of age on HRV and BRS were investigated using linear regression analysis (Table S2). In the 20–79-year age group, both HRV indexes (i.e., RR, HF and LF) and BRS indexes (i.e., BrrLF, BrrHF, BrrA, and BrrD) were negatively associated with age during nREM; specifically, this correlation was moderate for all the HRV indexes and BrrLF but strong for BrrHF, BrrA, and BrrD. Furthermore, during both wakefulness and nREM in the same age group, a negative linear relationship was noted BRS indexes (BrrLF, BrrHF, BrrA, and BrrD) and age; specifically, this correlation was moderate for BrrLF but strong for BrrHF, BrrA, and BrrD.

**Table S1.** Comparisons of HRV in Different Consciousness States Among Age Groups Using Wilcoxon Signed-Rank Test

|  | | **RR(ms)** | **HF[ln(ms^2^)]** | **LF[ln(ms^2^)]** | **LF/HF[ln(ratio)]** | **LF%(nu)** |
| --- | --- | --- | --- | --- | --- | --- |
| ***20-29*** years | AW | 932.31±122.74 | 6.56±0.73 | 7.16±0.62 | 0.60±0.61 | 63.35±12.90 |
|  | nREM | 971.99±108.33 | 6.60±0.83 | 6.69±0.58 | 0.04±0.61 | 52.00±13.90 |
|  | *p* | 0.157 | 0.983 | 0.048^*^ | 0.035^*^ | 0.035^*^ |
| ***30-49*** years | AW | 906.80±133.50 | 5.53±1.00 | 6.15±0.59 | 0.62±0.88 | 63.54±18.93 |
|  | nREM | 937.60±150.54 | 5.98±1.10 | 6.31±0.75 | 0.32±0.74 | 57.40±17.06 |
|  | *p* | 0.168 | 0.007^*^ | 0.108 | 0.082 | 0.072 |
| ***50-69*** years | AW | 936.35±132.06 | 4.79±0.93 | 5.68±0.92 | 0.90±0.55 | 69.89±10.54 |
|  | nREM | 1007.02±92.23 | 5.14±0.84 | 5.74±0.98 | 0.60±0.79 | 62.77±15.64 |
|  | *p* | 0.157 | 0.170 | 0.845 | 0.215 | 0.145 |
| ***70-79*** years | AW | 980.83±207.99 | 4.58±0.61 | 5.79±1.09 | 1.20±0.77 | 74.97±16.08 |
|  | nREM | 912.45±102.89 | 5.13±0.71 | 5.55±0.83 | 0.42±0.61 | 59.38±13.33 |
|  | *p* | 0.499 | 0.237 | 0.866 | 0.028^*^ | 0.028^*^ |

Mean ± SD
RR, R-R intervals; HF, high-frequency; LF low-frequency; LF/HF, LF-to-HF ratio; LF%, normalized LF; AW, awake; nREM, non-rapid eye
movement sleep

**p*<0.05

**Table S2.** Correlations of Age With HRV and BRS Values in Different Consciousness States Across Different Age Groups Using Spearman’s Rank Correlation Coefficients

|  | **AW** | | |  | **nREM** | | |
| --- | --- | --- | --- | --- | --- | --- | --- |
|  | *20-49* years | *50-79* years | *20-79* years |  | *20-49* years | *50-79* years | *20-79* years |
| **RR (ms)** | - | - | - |  | - | -0.48^*^ | - |
| **HF [ln(ms^2^)]** | -0.49^*^ | - | -0.65^*^ |  | -0.34^*^ | - | -0.58^*^ |
| **LF [ln(ms^2^)]** | -0.56^*^ | - | -0.59^*^ |  | - | - | -0.48^*^ |
| **LF/HF[ln(ratio)]** | - | - | 0.26^*^ |  | 0.38^*^ | - | 0.29^*^ |
| **LF% (nu)** | - | - | 0.28^*^ |  | 0.35^*^ | - | 0.27^*^ |
| **BrrLF (ms/mmHg)** | -0.43^*^ | - | -0.59^*^ |  | - | -0.50^*^ | -0.53^*^ |
| **BrrHF(ms/mmHg)** | - | -0.70^*^ | -0.50^*^ |  | - | - | -0.65^*^ |
| **BrrA (ms/mmHg)** | -0.44^*^ | -0.60^*^ | -0.55^*^ |  | -0.38^*^ | - | -0.66^*^ |
| **BrrD (ms/mmHg)** | - | - | -0.46^*^ |  | -0.37^*^ | - | -0.64^*^ |

RR, averaged means of the R-R intervals; HF, high-frequency; LF low-frequency; LF/HF, LF-to-HF ratio; LF%, normalized LF; BrrHF/BrrLF, the magnitude of the MAP-R-R intervals transfer functions; BrrA/BrrD, the slopes of mean arterial pressure-R-R intervals linear regressions; AW, awake; nREM, non-rapid eye movement

**p*<0.05
